# Improving the accuracy and resolution of neutron crystallographic data by three-dimensional profile fitting of Bragg peaks in reciprocal space

**DOI:** 10.1107/S2059798318013347

**Published:** 2018-10-29

**Authors:** Brendan Sullivan, Rick Archibald, Patricia S. Langan, Holger Dobbek, Martin Bommer, Robert L. McFeeters, Leighton Coates, Xiaoping Wang, Franz Gallmeier, John M. Carpenter, Vickie Lynch, Paul Langan

**Affiliations:** a Neutron Scattering Division, Neutron Sciences Directorate, Oak Ridge National Laboratory, 1 Bethel Valley Road, Oak Ridge, TN 37831, USA; b Computer Science and Mathematics Division, Computing and Computational Sciences Directorate, Oak Ridge National Laboratory, 1 Bethel Valley Road, Oak Ridge, TN 37831, USA; c Institut für Biologie, Humboldt-Universität zu Berlin, Philippstrasse 13, Leonor Michaelis Haus, 10115 Berlin, Germany; d Department of Chemistry, University of Alabama in Huntsville, 301 Sparkman Drive, Huntsville, AL 35899, USA; e Neutron Technologies Division, Neutron Sciences Directorate, Oak Ridge National Laboratory, 1 Bethel Valley Road, Oak Ridge, TN 37831, USA

**Keywords:** neutron crystallography, integration, profile fitting

## Abstract

Neutron crystallography is a powerful technique for directly visualizing the locations of H atoms in biological macromolecules. This information has provided key new insights into enzyme mechanisms, ligand binding and hydration. However, despite the importance of this information, the application of neutron crystallography in biology has been limited by the relatively low flux of available neutron beams and the large incoherent neutron scattering from hydrogen, both of which contribute to weak diffraction data with relatively low signal-to-background ratios. A method has been developed to fit weak data based on three-dimensional profile fitting of Bragg peaks in reciprocal space by an Ikeda–Carpenter function with a bivariate Gaussian. When applied to data collected from three different proteins, three-dimensional profile fitting yields intensities with higher correlation coefficients (CC_1/2_) at high resolutions, decreased *R*_free_ factors, extended resolutions and improved nuclear density maps. Importantly, additional features are revealed in nuclear density maps that may provide additional scientific information. These results suggest that three-dimensional profile fitting will help to extend the capabilities of neutron macromolecular crystallography.

## Abstract 2

It is demonstrated that using three-dimensional profile fitting of Bragg peaks increases the accuracy and resolution of neutron crystallographic data collected from proteins and reveals new features in nuclear density maps calculated from these data.

## 1. Introduction   

Neutron crystallography can provide structural, chemical and functional information on biological macromolecules that is difficult or impossible to obtain using other techniques (Blakeley *et al.*, 2008 ▸). One of its main advantages is the ability to directly visualize hydrogen (H) or deuterium (D) atoms at modest resolutions of around 2.0–2.5 Å (Bacik *et al.*, 2017 ▸; Kwon *et al.*, 2016 ▸; Casadei *et al.*, 2014 ▸; Coates *et al.*, 2008 ▸; Wan *et al.*, 2015 ▸; Chen & Unkefer, 2017 ▸). Despite its potential to elucidate the molecular mechanisms behind a wealth of phenomena (Langan *et al.*, 2018 ▸; Schaffner *et al.*, 2017 ▸), the application of neutron crystallography remains limited by the relatively weak intensity of available neutron beams and the high neutron scattering background arising from incoherent scattering by hydrogen within the sample (O’Dell *et al.*, 2016 ▸). While more powerful beamlines and advances in sample preparation have helped to address these challenges, there are also opportunities to develop more advanced computational tools to improve the accuracies of the measured neutron crystallographic data and of the resulting refined structures. Previously, we have developed new computational tools for joint X-ray and neutron refinement that result in more accurate structures (Afonine *et al.*, 2010 ▸). In this work, we focus on a new computational tool to increase the accuracy of the neutron crystallographic data.

One existing approach to integrating neutron Bragg peaks is to use peak-minus-background integration methods. These integration schemes sum events from a pre-defined volume centered at the peak and subtract the local background, which is determined by summing events from a separate, nearby volume with appropriate geometric scaling. While these schemes have proven to be successful, they face several critical disadvantages. Firstly, they may not appropriately account for the asymmetric peak shape at pulsed neutron sources. Neutron Bragg peaks from instruments with pulsed, moderated sources have a long tail on the high time-of-flight (TOF) end which is difficult to distinguish from background, resulting in either a decreased signal-to-noise ratio (with a generous peak-volume definition) or artificially decreased intensities (when this tail is considered to be background). In addition, they demand very precise knowledge of the location of each peak. For large unit-cell experiments in particular, being only a few pixels off can decrease the integrated intensity by factors of up to 50% with aggressive integration schemes. Using peak-minus-background integration, peaks that fall on or near detector edges may not be integrated accurately. In the case of a standard data set collected on the MaNDi beamline at the Spallation Neutron Source (SNS; Coates *et al.*, 2015 ▸), integration errors arising from peaks near detector edges may affect as many as one fifth of the peaks. Finally, as scientifically pertinent problems continue to demand higher resolution and the analysis of larger unit cells (Azadmanesh *et al.*, 2017 ▸), it becomes more difficult to quantify peak intensity as peaks become closer to each other and eventually overlap.

To address these issues, profile fitting has historically been employed. While analytical consideration of single-crystal Bragg peak intensities was first given serious consideration in 1962, Diamond was the first to demonstrate increased crystallographic data quality as a result of profile fitting (Alexander & Smith, 1962 ▸; Diamond, 1969 ▸). A decade later, profile fitting was extended to large unit cells using the ‘oscillation method’ (Rossmann, 1985 ▸; Harrison *et al.*, 1985 ▸) and has since been developed further (Pavese & Artioli, 1996 ▸; Leslie, 2006 ▸; Kabsch, 2006 ▸). While these techniques are appropriate for monochromatic X-ray and neutron Bragg peaks, planned user programs at pulsed neutron sources such as the European Spallation Source (ESS), Lund, Sweden and the Second Target Station at SNS, Oak Ridge, USA will enable the widespread use of TOF techniques. To maximize the effectiveness of experiments at current and future pulsed neutron sources, it is imperative to have algorithms that exploit the information provided by TOF profiling.

Crystallography beamlines at modern pulsed neutron sources use time-resolved area detectors to record diffracted neutrons. Recently, there have been a handful of proposals to fit TOF profiles to integrate peaks. Yano *et al.* (2016 ▸) demonstrated that profile fitting provides improved model structures from protein data. To carry out their profile fitting, the authors fitted the observed profiles to a Gaussian profile convolved with two back-to-back exponentials that phenomenologically describe the profiles. This is similar to the functional form proposed by Gutmann (2017 ▸), who noted that it describes the peak asymmetry arising from the tail well. The first report to examine fitting in reciprocal space (Schultz *et al.*, 2014 ▸) demonstrated decreased *R* factors using peaks integrated along the TOF profile compared with peak-minus-background integration. A complete description of the peak, however, must be three-dimensional to account for the two detector spatial dimensions and the TOF. Equivalently, these three dimensions can be expressed in reciprocal space. A preliminary report (Tomoyori & Tamada, 2016 ▸) suggested that three-dimensional profile fitting will be beneficial to data quality, but examined only a handful of peaks in detector space.

Here, we present an algorithm for integrating Bragg peaks by three-dimensional profile fitting in reciprocal space. The primary objective of this work is to improve data quality through more accurate integration of weak peaks and peaks that are partially recorded at the edge of detectors. However, we expect that three-dimensional profile fitting will also benefit the deconvolution of any overlapping peaks. After describing the algorithm in detail, we compare its performance with standard spherical integration schemes using three complete representative data sets collected on the MaNDi beamline. Two data sets are perdeuterated and one is H/D-exchanged, demonstrating the effectiveness of this technique for both types of samples. It is shown that profile fitting yields comparable merging *R* values for protein data sets yet, of particular interest, produces a significantly increased CC_1/2_ at high resolutions (Karplus & Diederichs, 2012 ▸). To assess the accuracy of each integration method, we carry out refinements of models from X-ray data against peaks from each integration method. In each case examined, profile fitting yields *R*_free_ factors demonstrating an increased accuracy from profile fitting. The first data set, perdeuterated E166Q β-lactamase mutant, shows a decrease in *R*_free_ of 2.3% at 1.89 Å resolution. The second data set, H/D-exchanged PsbO (an extrinsic subunit of photosystem II), shows a decrease in *R*_free_ of 2.3% at 2.2 Å resolution. The third data set, perdeuterated *Pseudomonas aeruginosa* peptidyl-tRNA hydrolase 1 (PaPth1), shows a decrease in *R*_free_ of 2.7% from initial refinement at 2.60 Å resolution. The increased resolution in data sets such as that of PaPth1 makes it possible to better visualize important features such as water molecules. Finally, the resulting nuclear density maps from each integration method are compared. Reflective of their decreased *R*_free_ values, nuclear density maps refined against profile-fitted intensities show better agreement with the atomic model. Given these results, it is clear that three-dimensional profile fitting has the potential to advance the capabilities of neutron crystallography.

## 2. Methods   

### 2.1. Data collection   

For initial testing, strong peaks from a scolecite data set recorded on the TOPAZ beamline at SNS, Oak Ridge, USA (Jogl *et al.*, 2011 ▸) were used. Protein data that contained many considerably weaker peaks were collected on the MaNDi beamline (Coates *et al.*, 2015 ▸). The protein data-collection strategy was optimized using the *CrystalPlan* package (Zikovsky *et al.*, 2011 ▸) and the numbers of orientations recorded are presented in Tables 1 ▸, 2 ▸ and 3 ▸. Crystallization of the E166Q β-lactamase mutant was carried out as described in Tomanicek *et al.* (2010 ▸), while PsbO was crystallized as described in Bommer *et al.* (2017 ▸). Crystallization of PaPth1 was achieved as described in McFeeters *et al.* (2016 ▸).

### 2.2. Moderator characterization by Monte Carlo simulations   

Neutron emission from the decoupled poisoned hydrogen moderator as viewed by the TOPAZ and MaNDi beamlines was simulated using *MCSTAS* (Nielsen & Lefmann, 2000 ▸) as described in Gallmeier (2010 ▸). Briefly, Monte Carlo simulations of the moderator output were fitted to the Ikeda–Carpenter (IC) function (Ikeda & Carpenter, 1985 ▸),
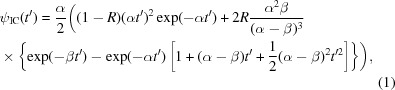
where ψ_IC_ is the intensity of neutrons from the moderator, α and β are energy-dependent constants, *R* is the energy-dependent ratio of slow to fast neutrons from the moderator and *t*′ = *t* − *t*_0_ > 0. This fit was performed for 141 logarithmically spaced energies ranging from 1 × 10^−5^ to 100 eV and the value of each parameter at each energy was fitted to a fourth-order Padé approximant. These values were used as an initial guess for fitting TOF profiles using the IC function.

### 2.3. Data reduction for profile fitting and strong peaks   

**Figure 1:**
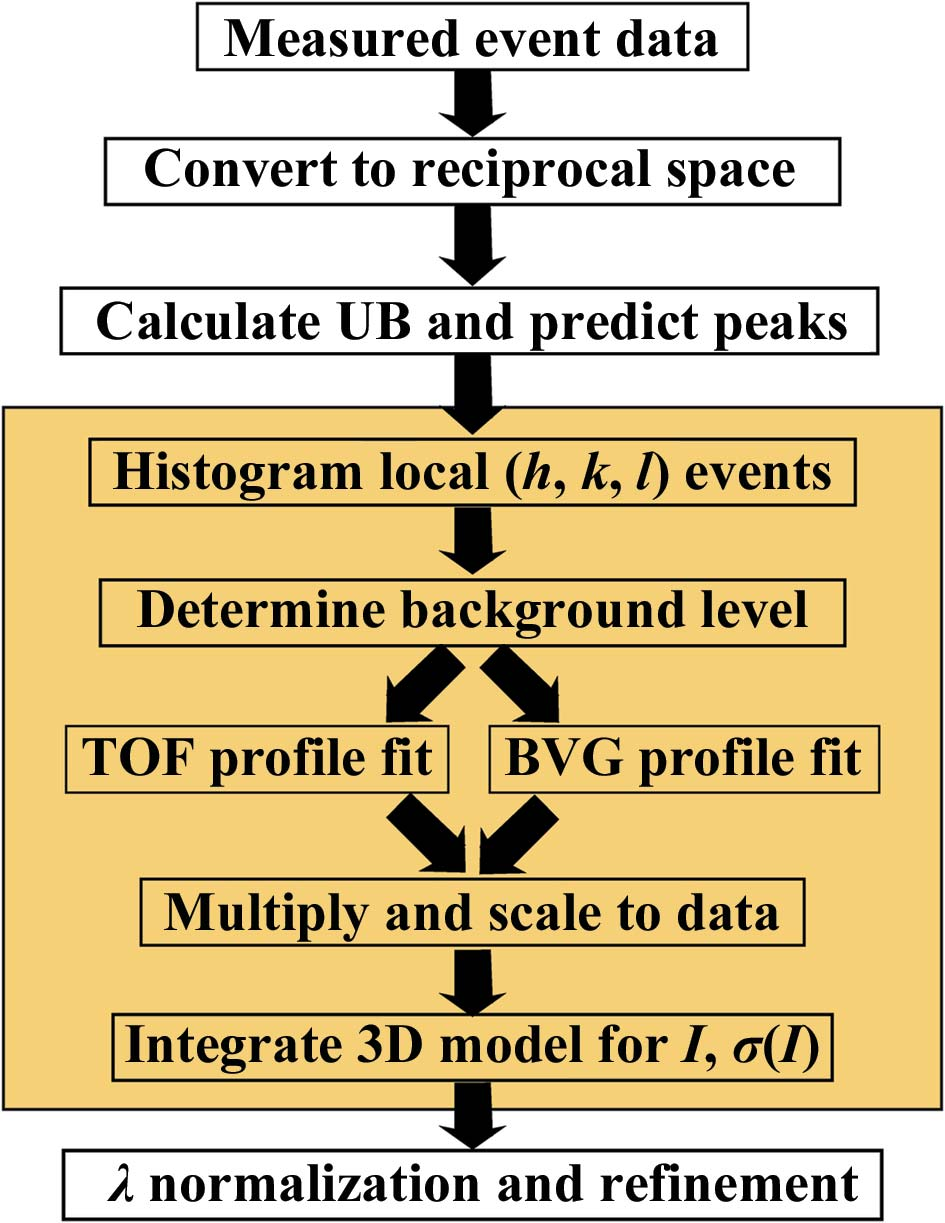
Flowchart showing the peak integration scheme for three-dimensional profile fitting. The steps in the yellow box are performed for each peak in a data set. Note that while predicted peak locations are used for initial guesses, the peak position is not restricted to its predicted location. The time-of-flight (TOF) and bivariate Gaussian (BVG) fits (§[Sec sec2.3]2.3) were performed separately to computationally simplify fitting all three dimensions. These two fits are then projected to three dimensions and multiplied together to generate the peak shape. This peak shape is then scaled to the observed data and background is added to create the model peak.

The integration scheme was tested using the *Mantid* framework (Arnold *et al.*, 2014 ▸), which allows the quick conversion of recorded event data to reciprocal space. First, an orientation matrix (**UB** matrix) is determined from several hundred bright peaks in reciprocal space. Given the **UB** matrix, the locations of all observable peaks were predicted using the *PredictPeaks* algorithm in *Mantid*. For the samples and resolutions presented in this work, the peaks did not overlap, as verified by ensuring that all integrated peaks were separated by at least the outer radius of the background used for spherical integration (§[Sec sec2.6]2.6). The procedure for each predicted peak is illustrated in the yellow box in Fig. 1 ▸.

**Figure 2:**
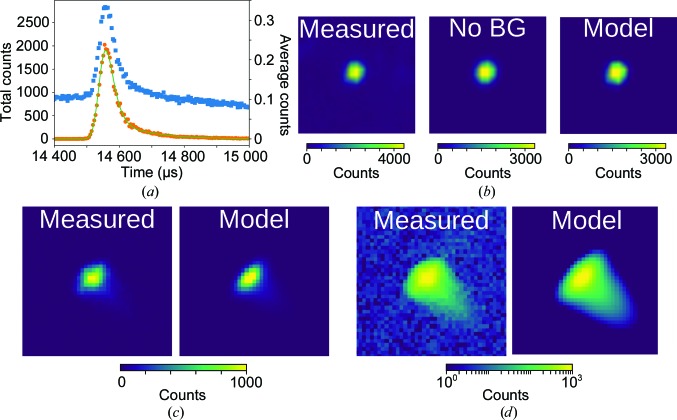
Peak and model for a very strong peak recorded on the TOPAZ beamline. (*a*) The TOF profile before (blue squares) and after (orange circles) background removal. The IC fit (green) nicely fits the high-TOF tail. (*b*) The bivariate normal fit to the non-TOF coordinates φ*az* and 2θ. The left box is a two-dimensional histogram before background removal, the middle box (‘No BG’) is made by summing only the voxels in reciprocal space used for fitting, and the right box (‘Model’) shows the fit to the data. (*c*, *d*) A slice of three-dimensional *q* space along *q_z_* of the peak as measured and the same slice of the three-dimensional model of the peak shown on a linear scale (*c*) and a logarithmic scale (*d*) to accentuate the head and tail, respectively.

**Figure 3:**
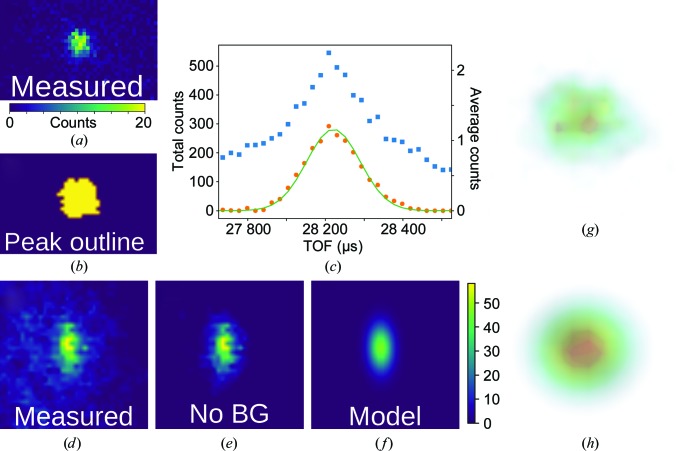
Example of background removal for a strong protein peak. (*a*) Slice of the peak as viewed from the *q_z_* axis. (*b*) The pixels from the slice in (*a*) which were determined to constitute the peak are shown in yellow. Pixels deemed background are shown in purple. (*c*) TOF spectra created from the entire histogrammed region (blue squares) and only voxels considered to be in the peak (orange circles). The fit to the IC profile is shown in green. (*d*, *e*, *f*) Angular histograms in 2θ and *q*φ*az* showing the whole peak (*d*), the background-removed peak (*e*) and the fit of the background-removed peak to a bivariate Gaussian distribution (*f*). (*g*) Three-dimensional volume rendering of the recorded peak. (*h*) Three-dimensional volume rendering of the three-dimensional model of the peak shown in (*g*).

For each peak with index **h** = (*h*, *k*, *l*), a histogram of recorded events from (*h* − η, *k* − η, *l* − η) to (*h* + η, *k* + η, *l* + η) is generated in reciprocal space. η is a parameter that determines how large a volume in reciprocal space is considered for background removal. In practice, this parameter can be varied in the range ∼0.2–0.5 with little effect on the resulting intensities. For the current work η = 0.25 was used. From this histogram, the background must be differentiated from the peak signal. To determine the appropriate background threshold, a nearest-neighbors smoothed histogram is generated. The threshold above which voxels (three-dimensional ‘pixels’ in reciprocal space) will be included in the peak will be determined from this smoothed histogram. Given that the energy of each peak is known and that emission from the moderator has been characterized by Monte Carlo simulations (§[Sec sec2.2]2.2), the expected TOF profile of each peak is known and only needs to be scaled for the number of neutrons. Thus, to determine the background threshold, it is sufficient to fit this expected profile to the resulting TOF profile at each background level until a satisfactory profile is found (χ^2^ ≃ 1). To achieve this, the TOF profile is generated by creating a histogram of events binned by TOF (TOF ∝ *L*sin(θ)/|**q**|), effectively summing the remaining two directions. This profile is fitted to the Ikeda–Carpenter function, ψ_IC_, convolved with a Gaussian and a top-hat function to account for detector broadening and finite proton-pulse duration, respectively. This is illustrated in Figs. 2 ▸(*a*) and 3 ▸(*c*), which show the TOF profile both before (blue) and after (orange) background subtraction. The background level is taken as the intensity with which the TOF profile is best described by the predicted TOF profile. These voxels (for example the slice shown in Fig. 3 ▸ *b*) are used to construct the three-dimensional model of the peak.

To generate the full three-dimensional profile, it is natural to consider the reciprocal-space histogram in spherical co­ordinates **q**(*q_x_*, *q_y_*, *q_z_*) → **q**(*q_r_*, *q*_φ*az*_, *q*_2θ_) as 1/*q_r_* ≃ TOF and *q*_φ*az*_ and *q*_2θ_ are described by a bivariate Gaussian distribution ψ_BVG_, where φ_*az*_ denotes the azimuthal coordinate (in the *xy* plane) and 2θ is the standard scattering angle coordinate (angle from the *z* axis). The angular distribution is fitted to a two-dimensional histogram in φ_*az*_ and 2θ, effectively summing *q_r_* (Figs. 2 ▸ *b* and 3 ▸ *f*). ψ_IC_ and ψ_BVG_ at this point are effectively independent probability distributions. Incorporating a scale factor, *A*, and a constant background term, *B*, the resulting three-dimensional model, ψ, is given by their product: ψ = *A*(ψ_IC_ × ψ_BVG_) + *B*, where *A* and *B* are determined by a least-squares fit to the three-dimensional event histogram in reciprocal space. Generating the model in reciprocal space, which scales linearly with *q* to provide an undistorted view of the three-dimensional peak profile, allows discretization at the level of instrument resolution rather than by generating thick slices, minimizing quantification error. A three-dimensional rendering of a peak and its model are shown in Figs. 3 ▸(*g*) and 3 ▸(*h*), while two-dimensional slices are shown in Figs. 2 ▸(*c*), 2 ▸(*d*) and 4 ▸(*b*). For completeness, it should be noted that this three-dimensional model is generated from a (2 + 1)-dimensional fit to simplify the least-squares optimization from a computational point of view. In practice, no difference was found between these fits and full three-dimensional profile fits.

### 2.4. Profile fitting for weak peaks and peaks on detector edges   

**Figure 4:**
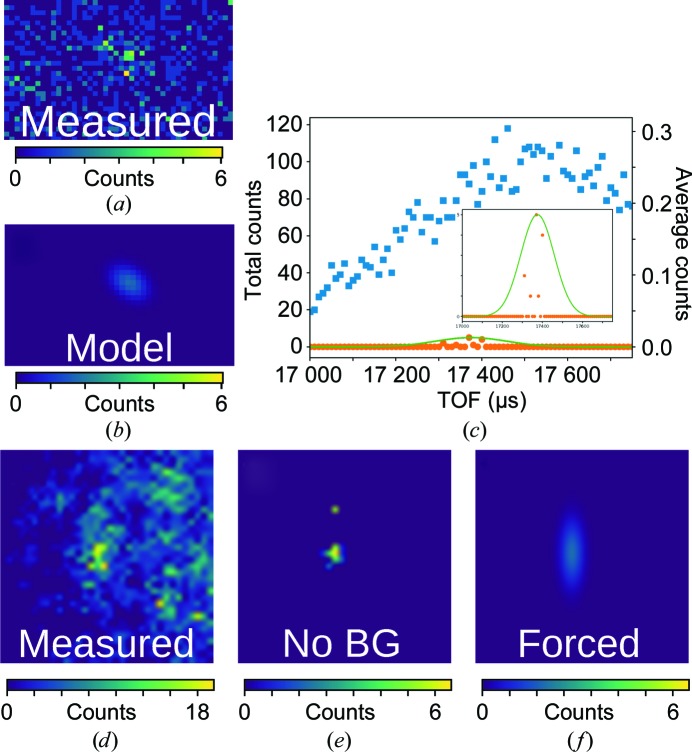
Three-dimensional profile fit of a weak peak. (*a*) A slice of *q_z_* for the peak. (*b*) The resulting three-dimensional model of the same slice. (*c*) The uncorrected (blue squares) and background-corrected (orange circles) TOF profiles with the optimal fit (green). The inset is zoomed in on the peak. (*d*, *e*, *f*) Angular histogram of the peak showing the raw histogram (*d*), the background-removed peak (*e*) and the profile of the forced nearest-neighbor peak used to construct the model (*f*).

**Figure 5:**
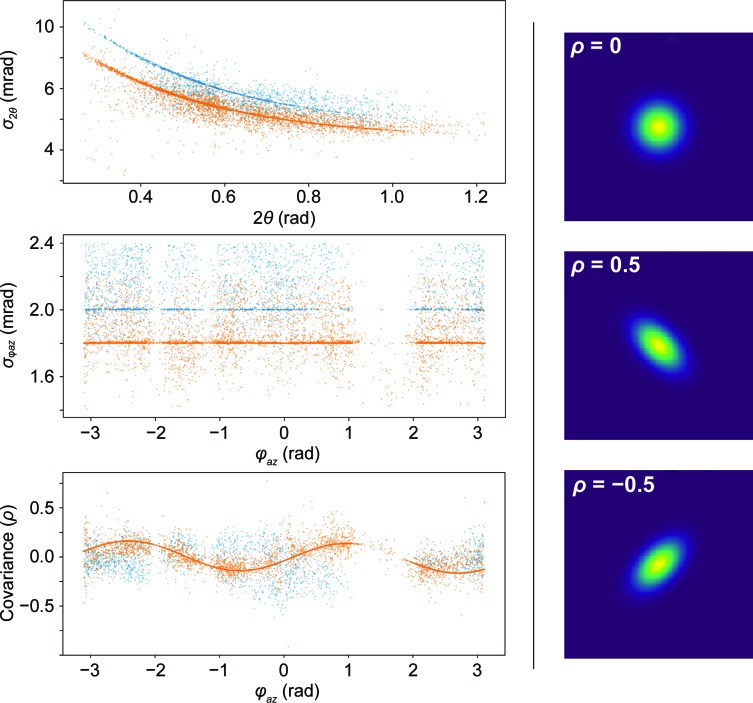
Left: scatter plots of parameters for ψ_BVG_ for the strong-peaks library from the β-lactamase E166Q (blue) and PsbO (orange) data sets. In both cases, peaks become smaller with increased scattering angle and remain relatively constant in size as a function of the azimuthal angle. The orientation of each peak is determined by the covariance, which oscillates with the azimuthal angle. Note that both data sets contain a strongly oscillating covariance, but only the PsbO curve is visible because they are overlaid. Right: three model bivariate Gaussians with different covariance (ρ) values. These demonstrate how covariance defines peak shape, which changes with φ_*az*_. Here, 2θ denotes the standard scattering angle (from the *z* axis) and the azimuthal angle, φ_*az*_, is the angle in the *xy* plane.

While the procedure described in §[Sec sec2.3]2.3 works well for strong peaks, it is expected that profile fitting will most benefit the integration of weak peaks where the background and peak are nearly indistinguishable. An example of such a peak is shown in Fig. 4 ▸(*a*). While the TOF direction can still be fitted using the moderator characterization (Fig. 4 ▸ *c*), there are too few counts to create a fittable angular histogram (Fig. 4 ▸ *d*). To circumvent this, and given that the profile of ψ_BVG_ changes slowly with φ_*az*_ and 2θ, the angular distribution ψ_BVG_ is assumed to be the same as a nearest neighbor in (*q*_φaz_, *q*_2θ_) from a library of strong peaks. For the work presented here, profiles were applied if the peak had fewer than 250 events (as determined by spherical integration). The strong-peak library was constructed from peaks containing more than 500 events (as determined by spherical integration) for each data set. The parameters defining peak shape for the strong-peaks libraries for E166Q β-lactamase and PsbO are shown in Fig. 5 ▸.

Since peaks near the detector edges may not be fully recorded, the profiles of the strong peaks can also be used to recover their intensity. In the present work, profiles were applied to edge peaks if the peak location was predicted to be 15 or fewer detector pixels from a detector edge. The merging statistics of peaks near the edge (between 1 and 15 pixels) are shown in Table 4 ▸.

### 2.5. Calculation of *I* and σ(*I*)   

Reliable refinement depends on accurate integration and error determination. Defining the observed number of neutrons in each voxel in reciprocal space as *N*_obs_, it is clear that for each voxel *N*_peak_ = *N*_obs_ − *N*_bg_, where *N*_peak_ and *N*_bg_ are the number of diffracted neutrons in the peak and background, respectively. The peak intensity *I* is then defined as *I* = 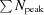. Following the same reasoning as Pflugrath (1999 ▸), the variance, σ^2^(*I*), of this intensity is just the sum of the associated variances. Assuming Poisson statistics (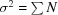), this can be expressed as
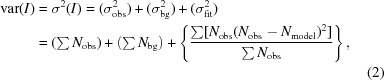
with the final term being the variance of the fit. At this point, quantification of peak intensity depends on how the volume of the peak is defined (*i.e.* which voxels are summed over) and how the background is determined. For the present work, the intensity is determined by summing the model intensities of voxels that are above 5% of the maximum value of *N*_model_. The background is assumed to be constant throughout the volume of the peak and is assumed to be the average number of neutrons in the (*h* − η, *k* − η, *l* − η) to (*h* + η, *k* + η, *l* + η) volume that is not considered a peak and is accessible with the detector coverage of the instrument.

### 2.6. Spherical integration of peak intensities   

For comparison with traditional integration, the same peak sets were analyzed using the standard integration and refinement protocol at MaNDi in parallel with profile-fitted peaks. The only difference between the two data sets is how they were integrated. Spherical integration was performed *via* the *Integrate­PeaksMD* algorithm in *Mantid*. Peaks from E166Q β-lactamase and PsbO were integrated with a radius of 0.021 Å^−1^ and the background shell was taken from 0.022 to 0.026 Å^−1^, while PaPth1 was integrated with a radius of 0.018 Å^−1^ with a background shell from 0.019 to 0.022 Å^−1^.

### 2.7. Analysis of integrated intensities and refinement details   

**Figure 6:**
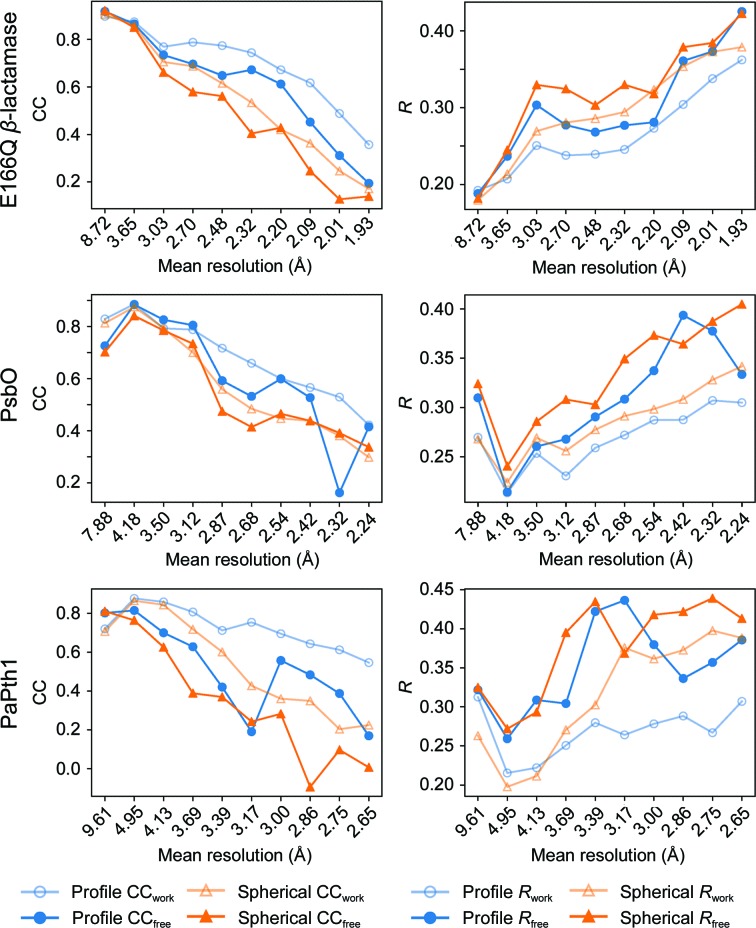
Shell-by-shell refinement statistics for each data set. CC values are shown on the left and *R* values on the right. From these plots it is clear that profile fitting has the largest effect on high-resolution data. Values are given in Supplementary Tables S6, S7 and S8.

After integration, protein peak intensities were scaled using *LAUENORM* from the *LAUEGEN* package (Campbell, 1995 ▸) and the merging statistics presented in Tables 1 ▸, 2 ▸ and 3 ▸ and Supplementary Tables S1, S2 and S3 were calculated using *PHENIX* (Adams *et al.*, 2010 ▸). For three-dimensional profile intensity data, data were rejected if χ^2^ of either the TOF, BVG or three-dimensional scaling fit was too large (χ^2^ > 50). Peaks with *I*/σ(*I*) < 1.0 from either profile fitting or spherical integration were rejected. Peaks were also removed if the peak center was one detector pixel from the edge. The statistics presented in these tables are discussed in Karplus & Diederichs (2012 ▸). To generate initial models for refinement, a Protein Data Bank (PDB) entry for the same protein generated from X-ray crystallography was used as a starting point. This model was aligned with the data using molecular replacement *via* *Phaser* (*phenix.phaser*). At this point, H or D atoms were added using *phenix.ready_set*. This model was refined using *phenix.refine* (Afonine *et al.*, 2012 ▸) against data sets integrated using each integration method. The peak data for refinement, including the selection of the working and testing data sets, are the same except for the intensities and uncertainties resulting from the integration method. For each protein, models were refined from the same initial model for nine iterations using *phenix.refine*. For E166Q β-lactamase, atomic positions, atomic *B* factors and occupancies were refined. Because refinement was performed at above 2 Å for PsbO and PaPth1, individual atomic positions were not refined, although rigid-body refinement was allowed. Overall *R* factors from refinements are shown in Tables 1 ▸, 2 ▸ and 3 ▸, while Fig. 6 ▸ and Supplementary Tables S6, S7 and S8 show CC and *R* from the refinements for each resolution shell.

To directly compare strong and weak peaks, merging statistics for E166Q β-lactamase and PsbO are presented in Tables S4 and S5. For these tables, peaks were separated by being either above or below the median *I*/σ(*I*) for each data set for each integration method. Merging statistics were calculated in *PHENIX* exactly as was performed for the whole data set. The same comparison is not presented for PaPth1 as the low number of peaks (<15 000 in the final data set) makes it difficult to directly compare the split peak sets.

## 3. Results   

### 3.1. Results for the E166Q β-lactamase mutant   

A summary of merging statistics and refinement statistics from refining the initial model of the E166Q β-lactamase mutant against peaks from each integration method is presented in Table 1 ▸. Shell-by-shell merging statistics are given in Supplementary Table S1, while Supplementary Table S4 shows the same statistics for weak and strong peaks separately. The most drastic difference in merging statistics is in Pearson’s correlation coefficient, CC_1/2_, at high resolutions (Supplementary Table S1). *I*/σ(*I*) is higher at low resolution and approaches *I*/σ(*I*) = 1 more quickly at high resolution.

**Figure 7:**
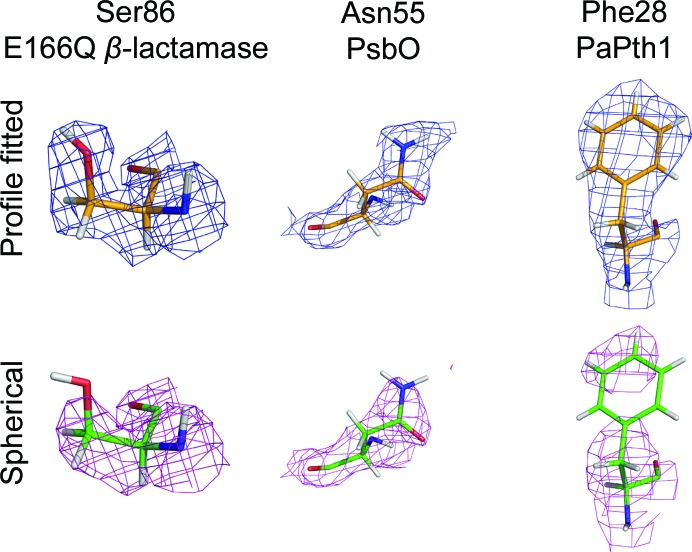
The 2*mF*_o_ − *DF*_c_ nuclear density maps for selected residues from each integration method. Left: Ser86 from the E166Q β-lactamase data set at 1.5σ shows that profile fitting recovers density for the OD of the carboxyl group. Middle: Asn55 from the PsbO data set and nearby water molecules at 1.1σ. With increased accuracy, profile fitting allows clear separation between the top water molecule and the residue. The water to the right, marked by crosses, is visible at lower σ (see Supporting information) Right: Phe28 from the PaPth1 data set at 1.9σ. It is clear that profile fitting recovers the nuclear density of the phenyl group. Densities at different σ levels are shown in the Supporting information.

Atomic positions were refined during the E166Q β-lactamase refinement. The models refined against profile-fitted and spherically integrated data differed by an r.m.s.d. of 0.09 Å. Shell-by-shell refinement statistics are shown in Fig. 6 ▸ and Supplementary Table S6. Overall, refinement against the known model yields increased CC and decreased *R* values, particularly in the medium- and high-resolution shells. Individual residues have several structural differences as a result of profile fitting. One such residue is highlighted in Fig. 7 ▸.

### 3.2. Results for PsbO   

A summary of merging and initial refinement statistics for PsbO is presented in Table 2 ▸, while Supplementary Tables S2 and S5 show shell-by-shell merging statistics. As with the E166Q β-lactamase mutant, three-dimensional profile fitting resulted in comparable overall merging *R* values and increased CC_1/2_, especially at high resolutions. The overall *I*/σ(*I*) values are again higher at low resolution and approach unity more quickly for profile-fitted peaks than spherically integrated peaks. Shell-by-shell refinement statistics are shown in Fig. 6 ▸ and Supplementary Table S7, which show increased CC values and decreased *R* values in the medium- and high-resolution shells.

### 3.3. Results for PaPth1   

A summary of merging and refinement statistics for PaPth1 is presented in Table 3 ▸, shell-by-shell merging statistics are shown in Supplementary Table S3 and shell-by-shell refinement statistics are presented in Fig. 6 ▸ and Supplementary Table S8.

### 3.4. Effect on nuclear density   

Better integration is expected to yield improved nuclear densities. Selected residues are shown in Fig. 7 ▸. One potential advantage of improved integration is the ability to resolve the location of additional atoms in amino-acid side chains, as illustrated by Ser86 in perdeuterated E166Q β-lactamase (Fig. 7 ▸, Supplementary Fig. S1). Density maps from profile-fitted intensities clearly resolve the OG atom (the top O atom in the images) and the bound D atom while maps derived from spherical integration are missing density for these atoms. Additionally, higher quality density maps allow atomic positions to be determined with higher certainty. Asn55 from the H/D-exchanged PsbO data set is shown in Fig. 7 ▸ and Supplementary Fig. S2. From inspection, it is clear that profile fitting results in better nuclear densities around the (top) ND2 atom and the bound DD21 and DD22 atoms. In addition, Phe28 from perdeuterated PaPth1 is shown in Fig. 7 ▸ and Supplementary Fig. S3. It is clear from inspection that the map from profile-fitted intensities better matches the perdeuterated phenyl ring. Clearer definition in features such as this is expected to enable the discovery of new structural details.

## 4. Discussion   

We have presented full three-dimensional profile fitting of entire neutron crystallographic data sets for the first time. In contrast to other recent profile fitting performed in detector space (Tomoyori & Tamada, 2016 ▸; Yano *et al.*, 2016 ▸; Gutmann, 2017 ▸), this integration is performed in reciprocal space. As has been argued previously (Schultz *et al.*, 2014 ▸), there are several convenient features of integrating in reciprocal space. Most notably, the peak shapes are straightforward to model. In particular, it is straightforward to isolate peaks at high resolutions. In reciprocal space these peaks maintain separation, and even with a unit cell as large as that of PsbO (∼200 Å) there are no obvious effects of peak overlap. The background can be straightforwardly assessed over a large volume of reciprocal space by considering (*h* − η, *k* − η, *l* − η) to (*h* + η, *k* + η, *l* + η), which aids the quantitation of high-resolution peaks over integration in detector space.

For these data sets, an overall increase in the average *I*/σ(*I*) was observed. Increases of approximately 25%, 40% and 15% were found for the E166Q β-lactamase mutant, PsbO and PaPth1, respectively. This difference is likely to be related to the background level of each data set. Profile fitting significantly reduces the amount of nonpeak volume integrated and so it is expected that increases in signal-to-noise will be seen in samples with higher background. It has been speculated (Tomoyori & Tamada, 2016 ▸) that there should be an increase of around 10% in signal-to-noise resulting from profile fitting, while noting that applying learned peak shapes to weak peaks may increase this further. This is fairly consistent with our reported *I*/σ(*I*) values. Of particular interest, these data sets exhibit increased *I*/σ(*I*) at low resolution and decreased *I*/σ(*I*) at high resolution. This is likely to be an artifact of a high *I*/σ(*I*) resulting from the spherical integration method. Experience has shown that *I*/σ(*I*) does not fall to unity at high resolutions when using the spherical integration method, and while *I*/σ(*I*) does not fall to 1.0 using profile fitting, it more quickly approaches the unity limit.

It is also interesting to consider the merging statistics. As a complete data set, profile fitting leads to comparable merging *R* values for all three data sets presented. At higher resolutions, though, the merging *R* values for profile-fitted peaks are slightly higher than those from spherically integrated intensities (Supplementary Tables S1, S2 and S3). These figures demonstrate that profile-fitted intensities have a higher spread at high resolution, though not necessarily that the intensities are less accurate. To assess accuracy, we refined models from X-ray data against peak sets which vary only in the integration method. Models refine better against profile-fitted intensities, demonstrating that the technique produces more accurate intensities. The Pearson’s correlation coefficient CC_1/2_ has been argued to be the most reliable indicator of the quality of a data set (Evans, 2011 ▸; Diederichs & Karplus, 2013 ▸). For all three data sets, substantially higher CC_1/2_ values are observed at higher resolution. This increased consistency is, of course, a consequence of the relative insensitivity of profile-fitted intensities to noise. In light of this, it is unsurprising that models refine better against profile-fitted data.

To further verify that profile fitting has the largest effect in more accurately integrating high-resolution data, shell-by-shell refinement statistics are presented in Fig. 6 ▸ and Supplementary Tables S6, S7 and S8. The CC_1/2_ and *R* values show that data–model agreement predominantly increases at medium and high resolutions. Taken together, these results strongly suggest that profile fitting more accurately integrates peaks for model refinement by accurately integrating high-resolution/weak peaks. The increase in CC_1/2_ is especially noticeable when comparing strong peaks with weak peaks. Supplementary Tables S4 and S5 compare peak sets which have been split into high and low *I*/σ(*I*). When considering the E166Q β-lactamase data set (Supplementary Table S4), high-resolution peaks have a CC_1/2_ above 0.19 in the outermost shells for profile-fitted peaks, while spherically integrated peaks quickly fall to CC_1/2_ < 0.1. PsbO, which overall has a higher *I*/σ(*I*), shows similar results (Supplementary Table S5).

For weak peaks, ψ_BVG_ profiles in the non-TOF directions (φ_*az*_, 2θ) were determined from a library of strong peaks. The notion of applying profiles from a library of strong peaks dates back to the 1980s in neutron crystallography (Sjölin & Wlodawer, 1981 ▸; Wilkinson *et al.*, 1988 ▸) and has since proven to be beneficial in solving several protein structures. Of the X-ray structures deposited in the PDB, peak integration for macromolecular crystallography has been dominated by *XDS*, *MOSFLM*, *HKL* and d*TREK (Kabsch, 2010 ▸; Leslie, 2006 ▸; Otwinowski & Minor, 1997 ▸; Pflugrath, 1999 ▸). More recently, *DIALS* has been released to facilitate the development of new algorithms and to process data from increasingly high-throughput crystallography facilities (Winter *et al.*, 2018 ▸). While all of these packages use profile fitting to fit weak or incomplete peaks, *MOSFLM* and *HKL* integrate three-dimensional peaks by summing a series of two-dimensional images, a technique termed two-dimensional integration. *XDS*, d*TREK and *DIALS*, on the other hand, integrate a full three-dimensional model of the peak described as a three-dimensional Gaussian. The integration scheme described in this work is most similar to three-dimensional integration, except that the third dimension arises from TOF (rather than φ-slicing) and the functional form in the third dimension is an Ikeda–Carpenter function. The parameters defining peak shape from profile fitting are presented in Fig. 5 ▸, which shows the parameters for peaks with 0.4 mrad of the σ_*az*_ value of each data set. It is clear that the peak size decreases along the scattering direction with increasing scattering angle. In addition, the peak orientation, defined by the covariance ρ in reciprocal space, clearly depends on the azimuthal angle. It is also clear that the peak profile changes appreciably for different samples. While using the profile of the nearest neighbors yielded more accurate intensities, the observed trends suggest that peaks can be modeled using the resolution function of the instrument and sample parameters which may further increase accuracy. It is also conceivable that a machine-learning-based approach could be developed to more accurately predict peak profiles for weak peaks.

In addition to more accurately integrating weak peaks, profile fitting offers the opportunity to recover data near the edge of detectors. As an example, merging statistics of pixels near the edge for the E166Q β-lactamase data set are shown in Table 4 ▸. Of particular interest, the CC_1/2_ for the profile-fitted data resembles CC_1/2_ for the entire data set, while spherically integrated peaks have a CC_1/2_ that quickly falls to 0. In traditional integration workflows, these intensities would typically be discarded or included despite poor quantification. While all of the data sets analysed so far were recorded using SNS Anger camera detectors (Riedel *et al.*, 2015 ▸), the capability to recover edge intensities also has the potential to benefit the integration of data recorded on position-sensitive tube detectors, which have considerably more gaps in detector coverage.

This algorithm has been implemented in the *Mantid* (Arnold *et al.*, 2014 ▸) software package as the *IntegratePeaks­ProfileFitting* algorithm.

## Supplementary Material

Supplementary Tables and Figures.. DOI: 10.1107/S2059798318013347/mn5117sup1.pdf

## Figures and Tables

**Table 1:** Summary of merging statistics for spherical integration and three-dimensional profile fitting for the E166Q β-lactamase mutant Values in parentheses are for the highest resolution shell.

	Profile fitting	Spherical
Unit-cell parameters (Å, °)	*a* = *b* = 73.7, *c* = 99.8, α = β = 90, γ = 120	
Space group	*P*3_2_21	
No. of orientations	11	
No. of unique reflections	23633 (1970)	
Resolution range (Å)	14.77–1.89 (1.96–1.89)	
Multiplicity	4.87 (2.50)	
Completeness (%)	93.6 (79.7)	
Mean *I*/σ(*I*)	8.7 (2.5)	6.2 (2.9)
*R*_merge_ (%)	22.6 (31.6)	22.3 (26.6)
*R*_p.i.m._ (%)	9.3 (20.5)	9.4 (17.5)
CC_1/2_	0.941 (0.285)	0.948 (0.036)
*R*_work_	0.210	0.230
*R*_free_	0.257	0.280

**Table 2:** Summary of merging statistics for spherical integration and three-dimensional profile fitting for PsbO Values in parentheses are for the highest resolution shell.

	Profile fitting	Spherical
Unit-cell parameters (Å, °)	*a* = *b* = 56.0, *c* = 194.9, α = β = 90, γ = 120	
Space group	*P*6_1_22	
No. of orientations	12	
No. of unique reflections	9118 (868)	
Resolution range (Å)	13.98–2.20 (2.28–2.20)	
Multiplicity	8.29 (5.06)	
Completeness (%)	92.39 (92.54)	
Mean *I*/σ(*I*)	13.2 (3.8)	9.4 (4.2)
*R*_merge_ (%)	24.8 (31.1)	23.4 (27.9)
*R*_p.i.m._ (%)	7.5 (13.8)	7.2 (12.5)
CC_1/2_	0.948 (0.277)	0.965 (−0.018)
*R*_work_	0.262	0.276
*R*_free_	0.297	0.320

**Table 3:** Summary of merging statistics for spherical integration and three-dimensional profile fitting for PaPth1 Values in parentheses are for the highest resolution shell.

	Profile fitting	Spherical
Unit-cell parameters (Å, °)	*a* = *b* = 64.93, *c* = 156.52, α = β = 90, γ = 120	
Space group	*P*6_1_22	
No. of orientations	6	
No. of unique reflections	4985 (483)	
Resolution range (Å)	13.73–2.60 (2.69–2.60)	
Multiplicity	2.80 (2.28)	
Completeness (%)	76.90 (77.99)	
Mean *I*/σ(*I*)	6.5 (2.5)	5.6 (3.9)
*R*_merge_ (%)	22.5 (31.3)	23.1 (27,2)
*R*_p.i.m._ (%)	12.7 (21.1)	13.3 (18.7)
CC_1/2_	0.911 (0.228)	0.890 (−0.035)
*R*_work_	0.262	0.294
*R*_free_	0.336	0.363

**Table 4:** Summary of merging statistics for peaks 15 pixels or fewer from detector edges for the E166Q β-lactamase data set These peaks are a subset of those presented in Table 1 ▸. Values in parentheses are for the highest resolution shell (1.96–1.89 Å).

	Profile fitting	Spherical
Percentage of total reflections	23.9 (25.4)	
No. of unique reflections	15895 (946)	
Multiplicity	1.73 (1.32)	
*R*_p.i.m._ (%)	20.1 (27.6)	19.5 (23.0)
CC_1/2_	0.927 (0.147)	0.818 (0.021)

## References

[bb1] Adams, P. D., Afonine, P. V., Bunkóczi, G., Chen, V. B., Davis, I. W., Echols, N., Headd, J. J., Hung, L.-W., Kapral, G. J., Grosse-Kunstleve, R. W., McCoy, A. J., Moriarty, N. W., Oeffner, R., Read, R. J., Richardson, D. C., Richardson, J. S., Terwilliger, T. C. & Zwart, P. H. (2010). *Acta Cryst.* D**66**, 213–221. 10.1107/S0907444909052925 PMC2815670 20124702

[bb2] Afonine, P. V., Grosse-Kunstleve, R. W., Echols, N., Headd, J. J., Moriarty, N. W., Mustyakimov, M., Terwilliger, T. C., Urzhumtsev, A., Zwart, P. H. & Adams, P. D. (2012). *Acta Cryst.* D**68**, 352–367. 10.1107/S0907444912001308 PMC3322595 22505256

[bb3] Afonine, P. V., Mustyakimov, M., Grosse-Kunstleve, R. W., Moriarty, N. W., Langan, P. & Adams, P. D. (2010). *Acta Cryst.* D**66**, 1153–1163. 10.1107/S0907444910026582 PMC2967420 21041930

[bb4] Alexander, L. E. & Smith, G. S. (1962). *Acta Cryst.* **15**, 983–1004.

[bb5] Arnold, O., Bilheux, J.-C., Borreguero, J., Buts, A., Campbell, S. I., Chapon, L., Doucet, M., Draper, N., Ferraz Leal, R., Gigg, M., Lynch, V. E., Markvardsen, A., Mikkelson, D. J., Mikkelson, R. L., Miller, R., Palmen, K., Parker, P., Passos, G., Perring, T. G., Peterson, P. F., Ren, S., Reuter, M. A., Savici, A. T., Taylor, J. W., Taylor, R. J., Tolchenov, R., Zhou, W. & Zikovsky, J. (2014). *Nucl. Instrum. Methods Phys. Res. A*, **764**, 156–166.

[bb6] Azadmanesh, J., Trickel, S. R., Weiss, K. L., Coates, L. & Borgstahl, G. E. O. (2017). *Acta Cryst.* F**73**, 235–240. 10.1107/S2053230X17003508 PMC5379174 28368283

[bb7] Bacik, J.-P., Mekasha, S., Forsberg, Z., Kovalevsky, A. Y., Vaaje-Kolstad, G., Eijsink, V. G. H., Nix, J. C., Coates, L., Cuneo, M. J., Unkefer, C. J. & Chen, J. C.-H. (2017). *Biochemistry*, **56**, 2529–2532. 10.1021/acs.biochem.7b00019 28481095

[bb8] Blakeley, M. P., Ruiz, F., Cachau, R., Hazemann, I., Meilleur, F., Mitschler, A., Ginell, S., Afonine, P., Ventura, O. N., Cousido-Siah, A., Haertlein, M., Joachimiak, A., Myles, D. & Podjarny, A. (2008). *Proc. Natl Acad. Sci. USA*, **105**, 1844–1848. 10.1073/pnas.0711659105 PMC2538850 18250329

[bb9] Bommer, M., Coates, L., Dau, H., Zouni, A. & Dobbek, H. (2017). *Acta Cryst.* F**73**, 525–531. 10.1107/S2053230X17012171 PMC5619745 28876232

[bb10] Campbell, J. W. (1995). *J. Appl. Cryst.* **28**, 228–236.

[bb11] Casadei, C. M., Gumiero, A., Metcalfe, C. L., Murphy, E. J., Basran, J., Concilio, M. G., Teixeira, S. C. M., Schrader, T. E., Fielding, A. J., Ostermann, A., Blakeley, M. P., Raven, E. L. & Moody, P. C. E. (2014). *Science*, **345**, 193–197. 10.1126/science.1254398 25013070

[bb12] Chen, J. C.-H. & Unkefer, C. J. (2017). *IUCrJ*, **4**, 72–86. 10.1107/S205225251601664X PMC5331467 28250943

[bb13] Coates, L., Cuneo, M. J., Frost, M. J., He, J., Weiss, K. L., Tomanicek, S. J., McFeeters, H., Vandavasi, V. G., Langan, P. & Iverson, E. B. (2015). *J. Appl. Cryst.* **48**, 1302–1306.

[bb14] Coates, L., Tuan, H.-F., Tomanicek, S., Kovalevsky, A., Mustyakimov, M., Erskine, P. & Cooper, J. (2008). *J. Am. Chem. Soc.* **130**, 7235–7237. 10.1021/ja801269x PMC2607119 18479128

[bb15] Diamond, R. (1969). *Acta Cryst.* A**25**, 43–55. 10.1107/s0567739469000064 5799610

[bb16] Diederichs, K. & Karplus, P. A. (2013). *Acta Cryst.* D**69**, 1215–1222. 10.1107/S0907444913001121 PMC3689524 23793147

[bb17] Evans, P. R. (2011). *Acta Cryst.* D**67**, 282–292. 10.1107/S090744491003982X PMC3069743 21460446

[bb50] Gallmeier, F. X. (2010). *SNS Source Descriptions for Use with MCSTAS.* Tech. Rep. SNS106100200-TR0195-R00. Neutron Sciences Directorate, Oak Ridge National Laboratory.

[bb18] Gutmann, M. J. (2017). *Nucl. Instrum. Methods Phys. Res. A*, **848**, 170–173.

[bb19] Harrison, S. C., Winkler, F. K., Schutt, C. E. & Durbin, R. M. (1985). *Methods Enzymol.* **114**, 211–237. 10.1016/0076-6879(85)14021-8 4079768

[bb20] Ikeda, S. & Carpenter, J. M. (1985). *Nucl. Instrum. Methods Phys. Res. A*, **239**, 536–544.

[bb21] Jogl, G., Wang, X., Mason, S. A., Kovalevsky, A., Mustyakimov, M., Fisher, Z., Hoffman, C., Kratky, C. & Langan, P. (2011). *Acta Cryst.* D**67**, 584–591. 10.1107/S090744491101496X PMC3107055 21636899

[bb22] Kabsch, W. (2006). *International Tables for Crystallography*, Vol. F, edited by M. G. Rossmann & E. Arnold, pp. 218–225. Chester: International Union of Crystallography.

[bb23] Kabsch, W. (2010). *Acta Cryst.* D**66**, 125–132. 10.1107/S0907444909047337 PMC2815665 20124692

[bb24] Karplus, P. A. & Diederichs, K. (2012). *Science*, **336**, 1030–1033. 10.1126/science.1218231 PMC3457925 22628654

[bb25] Kwon, H., Basran, J., Casadei, C. M., Fielding, A. J., Schrader, T. E., Ostermann, A., Devos, J. M., Aller, P., Blakeley, M. P., Moody, P. C. E. & Raven, E. L. (2016). *Nature Commun.* **7**, 13445. 10.1038/ncomms13445 PMC5141285 27897163

[bb26] Langan, P. S., Vandavasi, V. G., Cooper, S. J., Weiss, K. L., Ginell, S. L., Parks, J. M. & Coates, L. (2018). *ACS Catal.* **8**, 2428–2437.

[bb27] Leslie, A. G. W. (2006). *Acta Cryst.* D**62**, 48–57. 10.1107/S0907444905039107 16369093

[bb28] McFeeters, H., Vandavasi, V. G., Weiss, K. L., Coates, L. & McFeeters, R. L. (2016). *Acta Cryst.* F**72**, 220–223. 10.1107/S2053230X16001813 PMC4774881 26919526

[bb29] Nielsen, K. & Lefmann, K. (2000). *Physica B*, **283**, 426–432.

[bb30] O’Dell, W. B., Bodenheimer, A. M. & Meilleur, F. (2016). *Arch. Biochem. Biophys.* **602**, 48–60. 10.1016/j.abb.2015.11.033 26592456

[bb31] Otwinowski, Z. & Minor, W. (1997). *Methods Enzymol.* **276**, 307–326. 10.1016/S0076-6879(97)76066-X 27754618

[bb32] Pavese, A. & Artioli, G. (1996). *Acta Cryst.* A**52**, 890–897.

[bb33] Pflugrath, J. W. (1999). *Acta Cryst.* D**55**, 1718–1725. 10.1107/s090744499900935x 10531521

[bb34] Riedel, R., Donahue, C., Visscher, T. & Montcalm, C. (2015). *Nucl. Instrum. Methods Phys. Res. A*, **794**, 224–233.

[bb35] Rossmann, M. G. (1985). *Methods Enzymol.* **114**, 237–280. 10.1016/0076-6879(85)14022-x 4079769

[bb36] Schaffner, I., Mlynek, G., Flego, N., Pühringer, D., Libiseller-Egger, J., Coates, L., Hofbauer, S., Bellei, M., Furtmüller, P. G., Battistuzzi, G., Smulevich, G., Djinović-Carugo, K. & Obinger, C. (2017). *ACS Catal.* **7**, 7962–7976. 10.1021/acscatal.7b01749 PMC5678291 29142780

[bb37] Schultz, A. J., Jørgensen, M. R. V., Wang, X., Mikkelson, R. L., Mikkelson, D. J., Lynch, V. E., Peterson, P. F., Green, M. L. & Hoffmann, C. M. (2014). *J. Appl. Cryst.* **47**, 915–921.

[bb38] Sjölin, L. & Wlodawer, A. (1981). *Acta Cryst.* A**37**, 594–604.

[bb39] Tomanicek, S. J., Blakeley, M. P., Cooper, J., Chen, Y., Afonine, P. V. & Coates, L. (2010). *J. Mol. Biol.* **396**, 1070–1080. 10.1016/j.jmb.2009.12.036 20036259

[bb40] Tomoyori, K. & Tamada, T. (2016). *J. Phys. Conf. Ser.* **762**, 012040.

[bb41] Wan, Q., Parks, J. M., Hanson, B. L., Fisher, S. Z., Ostermann, A., Schrader, T. E., Graham, D. E., Coates, L., Langan, P. & Kovalevsky, A. (2015). *Proc. Natl Acad. Sci. USA*, **112**, 12384–12389. 10.1073/pnas.1504986112 PMC4603456 26392527

[bb42] Wilkinson, C., Khamis, H. W., Stansfield, R. F. D. & McIntyre, G. J. (1988). *J. Appl. Cryst.* **21**, 471–478.

[bb43] Winter, G., Waterman, D. G., Parkhurst, J. M., Brewster, A. S., Gildea, R. J., Gerstel, M., Fuentes-Montero, L., Vollmar, M., Michels-Clark, T., Young, I. D., Sauter, N. K. & Evans, G. (2018). *Acta Cryst.* D**74**, 85–97. 10.1107/S2059798317017235 PMC5947772 29533234

[bb44] Yano, N., Yamada, T., Hosoya, T., Ohhara, T., Tanaka, I. & Kusaka, K. (2016). *Sci. Rep.* **6**, 36628. 10.1038/srep36628 PMC5131355 27905404

[bb45] Zikovsky, J., Peterson, P. F., Wang, X. P., Frost, M. & Hoffmann, C. (2011). *J. Appl. Cryst.* **44**, 418–423.

