# Supplementary material for: Improving the accuracy and resolution of neutron crystallographic data by three-dimensional profile fitting of Bragg peaks in reciprocal space
Source: Acta Crystallogr D Struct Biol. 2018 Oct 29;74(Pt 11):1085–95. doi: 10.1107/S2059798318013347 (PMC6213576; doi:10.1107/S2059798318013347)
Supplement: Supplementary file 1 [file d-74-01085-sup1.pdf]

STRUCTURAL
BIOLOGY

Volume 74 (2018)

Supporting information for article:

Improving the accuracy and resolution of neutron crystallographic data by three-dimensional profile fitting of Bragg peaks in reciprocal space

Brendan Sullivan, Rick Archibald, Patricia S. Langan, Holger Dobbek, Martin Bommer, Robert L. McFeeters, Leighton Coates, Xiaoping P. Wang, Franz Gallmeier, John M. Carpenter, Vickie Lynch and Paul Langan

Table S1 Shell-by-shell merging statistics for the E166Q β-lactamase mutant.

Resolution	Num Obs	Num Unique	Multiplicity	Completeness	Mean I	Mean I/σ(I)	R_{merge}	R_{meas}	R_{pim}	$CC_{1/2}$
**Profile Fitted**										
14.77-4.05	15762	2570	6.13	96.47	407.2	27.3	0.193	0.21	0.08	0.907
4.05-3.23	17395	2516	6.91	98.05	134.5	15.7	0.207	0.221	0.076	0.947
3.23-2.82	15365	2464	6.24	97.35	68.8	9.3	0.243	0.261	0.09	0.895
2.82-2.57	13466	2418	5.57	96.22	53	7.1	0.27	0.291	0.106	0.873
2.57-2.38	12779	2438	5.24	96.21	46.6	6.1	0.282	0.306	0.114	0.834
2.38-2.24	11088	2369	4.68	95.52	41.6	5.1	0.304	0.333	0.13	0.754
2.24-2.13	9679	2362	4.1	94.4	36.6	4.2	0.318	0.354	0.148	0.686
2.13-2.04	7953	2264	3.51	91.4	32.8	3.5	0.318	0.361	0.164	0.545
2.04-1.96	6737	2262	2.98	90.05	31.4	2.9	0.319	0.371	0.183	0.464
1.96-1.89	4929	1970	2.5	79.69	30.9	2.5	0.316	0.381	0.205	0.285
**14.77-1.89**	**115153**	**23633**	**4.87**	**93.59**	**92.6**	**8.7**	**0.226**	**0.246**	**0.093**	**0.941**
**Spherical Integration**										
14.77-4.05	15762	2570	6.13	96.47	740.2	20.2	0.182	0.199	0.077	0.92
4.05-3.23	17395	2516	6.91	98.05	242.1	11.2	0.224	0.241	0.084	0.938
3.23-2.82	15365	2464	6.24	97.35	129.1	6.8	0.269	0.29	0.103	0.798
2.82-2.57	13466	2418	5.57	96.22	98.2	5.5	0.279	0.303	0.113	0.678
2.57-2.38	12779	2438	5.24	96.21	85.4	4.9	0.292	0.319	0.123	0.514
2.38-2.24	11088	2369	4.68	95.52	77.5	4.4	0.292	0.323	0.131	0.314
2.24-2.13	9679	2362	4.1	94.4	72.2	3.9	0.289	0.325	0.142	0.206
2.13-2.04	7953	2264	3.51	91.4	67.1	3.5	0.279	0.32	0.15	0.157
2.04-1.96	6737	2262	2.98	90.05	63.6	3.2	0.266	0.313	0.159	0.11
1.96-1.89	4929	1970	2.5	79.69	62	2.9	0.266	0.322	0.175	0.036
**14.77-1.89**	**115153**	**23633**	**4.87**	**93.59**	**171.3**	**6.9**	**0.223**	**0.243**	**0.094**	**0.948**

Table S2 Shell-by-shell merging statistics for PsbO.

Resolution	Num Obs	Num Unique	Multiplicity	Completeness	Mean I	Mean I/σ(I)	R_{merge}	R_{meas}	R_{pim}	$CC_{1/2}$
**Profile Fitted**										
13.98-4.69	9191	962	9.55	87.3	705.4	37.6	0.216	0.228	0.069	0.912
4.69-3.74	11776	965	12.2	94.79	364.3	31.2	0.228	0.237	0.061	0.95
3.74-3.28	10606	939	11.29	93.43	168.4	16.9	0.273	0.284	0.072	0.867
3.28-2.98	9329	913	10.22	92.69	115.5	11.2	0.302	0.314	0.082	0.859
2.98-2.77	7856	907	8.66	93.03	86.3	7.8	0.304	0.319	0.09	0.799
2.77-2.61	6466	899	7.19	92.58	72.7	6	0.306	0.325	0.102	0.731
2.61-2.48	5698	884	6.45	92.18	66.8	5.2	0.31	0.333	0.115	0.631
2.48-2.37	5300	878	6.04	92.42	63.7	4.7	0.316	0.342	0.123	0.53
2.37-2.28	4965	903	5.5	93.58	61.3	4.3	0.309	0.337	0.125	0.478
2.29-2.20	4392	868	5.06	92.54	59.4	3.8	0.311	0.344	0.138	0.277
**13.98-2.20**	**75579**	**9118**	**8.29**	**92.39**	**182**	**13.2**	**0.248**	**0.261**	**0.075**	**0.948**
**Spherical Integration**										
13.98-4.69	9191	962	9.55	87.3	1402.6	23.4	0.204	0.215	0.066	0.934
4.69-3.74	11776	965	12.2	94.79	623.3	19.3	0.22	0.229	0.059	0.969
3.74-3.28	10606	939	11.29	93.43	294.1	11.2	0.264	0.275	0.072	0.909
3.28-2.98	9329	913	10.22	92.69	194.3	8.1	0.285	0.298	0.082	0.802
2.98-2.77	7856	907	8.66	93.03	148.1	6.4	0.302	0.319	0.096	0.504
2.77-2.61	6466	899	7.19	92.58	127.8	5.5	0.295	0.315	0.104	0.367
2.61-2.48	5698	884	6.45	92.18	115.2	5	0.297	0.321	0.114	0.269
2.48-2.37	5300	878	6.04	92.42	106.8	4.8	0.292	0.317	0.117	0.261
2.37-2.28	4965	903	5.5	93.58	99.4	4.4	0.286	0.312	0.117	0.125
2.29-2.20	4392	868	5.06	92.54	94.9	4.2	0.279	0.309	0.125	-0.018
**13.98-2.20**	**75579**	**9118**	**8.29**	**92.39**	**331.4**	**9.4**	**0.234**	**0.247**	**0.072**	**0.965**

Table S3 Shell-by-shell merging statistics for PaPth1.

Resolution	Num Obs	Num Unique	Multiplicity	Completeness	Mean I	Mean I/σ(I)	R_{merge}	R_{meas}	R_{pim}	$CC_{1/2}$
**Profile Fitted**										
13.73-5.49	1118	430	2.6	58.98	281.8	12.6	0.209	0.245	0.12	0.903
5.49-4.41	1656	497	3.33	72.87	254	13.9	0.205	0.236	0.109	0.893
4.41-3.86	1871	555	3.37	84.09	155	10.6	0.214	0.245	0.113	0.858
3.86-3.52	1811	565	3.21	85.22	81.3	6.9	0.231	0.272	0.135	0.733
3.52-3.27	1622	536	3.03	83.75	61	5.3	0.244	0.285	0.141	0.771
3.27-3.08	1392	527	2.64	81.71	47.3	4	0.253	0.303	0.161	0.682
3.08-2.93	1250	492	2.54	76.64	38.9	3.4	0.291	0.343	0.172	0.628
2.93-2.80	1175	481	2.44	75.75	33.2	3	0.287	0.347	0.187	0.48
2.80-2.69	1126	471	2.39	74.29	29.6	2.7	0.312	0.377	0.203	0.297
2.69-2.60	1115	489	2.28	77.99	25.8	2.5	0.313	0.381	0.211	0.228
**13.73-2.60**	**14136**	**5043**	**2.8**	**76.9**	**98.9**	**6.5**	**0.225**	**0.262**	**0.127**	**0.911**
**Spherical Integration**										
13.73-5.49	1118	430	2.6	58.98	596.2	8.7	0.187	0.22	0.11	0.913
5.49-4.41	1656	497	3.33	72.87	478	9.4	0.211	0.243	0.113	0.84
4.41-3.86	1871	555	3.37	84.09	321.2	7.5	0.233	0.267	0.123	0.753
3.86-3.52	1811	565	3.21	85.22	197.6	5.5	0.258	0.3	0.146	0.581
3.52-3.27	1622	536	3.03	83.75	157.4	4.8	0.26	0.304	0.15	0.481
3.27-3.08	1392	527	2.64	81.71	132	4.3	0.261	0.311	0.163	0.305
3.08-2.93	1250	492	2.54	76.64	119.5	4.1	0.27	0.32	0.164	0.175
2.93-2.80	1175	481	2.44	75.75	111.2	4	0.272	0.33	0.18	0.077
2.80-2.69	1126	471	2.39	74.29	105.4	4	0.273	0.331	0.18	0.002
2.69-2.60	1115	489	2.28	77.99	98	3.9	0.272	0.333	0.187	-0.035
**13.73-2.60**	**14136**	**5043**	**2.8**	**76.9**	**227.6**	**5.6**	**0.231**	**0.27**	**0.133**	**0.89**

Table S4 Shell-by-shell merging statistics for the E166Q β-lactamase mutant sorted by I/σ(I).

Resolution	Num Obs	Num Unique	Multiplicity	Completeness	Mean I	Mean I/σ(I)	R_{merge}	R_{meas}	R_{pim}	$CC_{1/2}$
**Profile Fitted – Low I/σ(I)**										
12.42-4.03	1732	772	2.24	29.06	904.4	2.9	0.304	0.368	0.202	0.243
4.03-3.22	3764	1448	2.6	56.67	871.1	3.1	0.268	0.322	0.172	0.211
3.22-2.82	5934	2006	2.96	79.16	814.1	3.2	0.275	0.327	0.171	0.326
2.82-2.56	6787	2149	3.16	85.62	749.5	3.2	0.287	0.337	0.171	0.318
2.56-2.38	7475	2260	3.31	89.86	726.5	3.2	0.296	0.346	0.173	0.372
2.38-2.24	7299	2287	3.19	91.96	695	3.0	0.309	0.362	0.182	0.302
2.24-2.13	7318	2296	3.19	91.88	656.1	2.9	0.317	0.371	0.186	0.283
2.13-2.04	6632	2227	2.98	90.09	625.3	2.8	0.31	0.366	0.189	0.288
2.04-1.96	5915	2210	2.68	88.61	612.8	2.5	0.312	0.375	0.201	0.257
1.96-1.89	4705	1978	2.38	79.5	618.1	2.3	0.308	0.377	0.212	0.197
12.42-1.89	57561	19633	2.93	77.88	708.6	2.9	0.299	0.354	0.183	0.308
**Profile Fitted – High I/σ(I)**										
14.42-4.06	14046	2346	5.99	88.36	9263.2	29.6	0.182	0.198	0.076	0.909
4.06-3.23	13521	2012	6.72	79.18	3447.5	18.7	0.184	0.197	0.068	0.948
3.23-2.83	9493	1567	6.06	62.36	2059.1	12.5	0.207	0.222	0.077	0.879
2.83-2.57	6682	1308	5.11	52.09	1746.3	10.2	0.223	0.242	0.089	0.815
2.57-2.39	5260	1154	4.56	46.05	1622	9.0	0.227	0.248	0.095	0.74
2.39-2.25	3807	936	4.07	37.58	1593.9	8.2	0.239	0.263	0.105	0.645
2.25-2.13	2381	710	3.35	28.69	1527.2	7.1	0.249	0.28	0.122	0.495
2.13-2.04	1324	473	2.8	19.24	1464	6.1	0.247	0.282	0.129	0.319
2.04-1.96	787	328	2.4	13.23	1473	5.6	0.259	0.299	0.143	0.337
1.96-1.89	290	167	1.74	6.77	1465.9	4.6	0.224	0.272	0.148	0.26
14.42-1.89	57591	11001	5.24	43.78	3640.3	15.3	0.193	0.209	0.078	0.935
**Spherical - Low I/σ(I)**										
10.71-4.01	2124	1042	2.04	39.35	1786.4	2.4	0.192	0.239	0.138	0.253
4.01-3.21	4670	1748	2.67	68.63	1271	2.7	0.198	0.237	0.127	0.171
3.21-2.81	6735	2207	3.05	87.75	1081.3	2.8	0.197	0.233	0.119	0.142
2.81-2.56	7362	2301	3.2	90.95	942	2.9	0.201	0.236	0.118	0.156
2.56-2.38	7677	2284	3.36	92.06	864.8	2.9	0.208	0.242	0.12	0.115

2.38-2.24	7392	2299	3.22	91.59	820.7	2.8	0.209	0.244	0.122	0.114
2.24-2.13	6833	2255	3.03	90.93	789.6	2.7	0.212	0.25	0.129	0.028
2.13-2.04	5883	2151	2.74	87.01	742.2	2.5	0.206	0.247	0.132	0.088
2.04-1.96	5083	2100	2.42	84.51	713.2	2.4	0.199	0.243	0.135	0.103
1.96-1.89	3812	1822	2.09	73.41	689.6	2.2	0.196	0.245	0.143	0.068
10.71-1.89	57571	20209	2.85	80.36	921.9	2.7	0.203	0.241	0.126	0.484
**Spherical - High I/σ(I)**										
14.42-4.06	13780	2457	5.61	92.33	10361.4	21.0	0.164	0.18	0.07	0.929
4.06-3.23	12722	2256	5.64	88.06	3737.6	11.9	0.183	0.197	0.071	0.94
3.23-2.83	8605	1950	4.41	77.88	2277.5	7.5	0.195	0.213	0.082	0.762
2.83-2.57	6149	1791	3.43	70.87	1830.6	5.9	0.188	0.211	0.09	0.577
2.57-2.39	4979	1688	2.95	67.47	1675.4	5.3	0.187	0.213	0.096	0.435
2.39-2.25	3723	1521	2.45	60.99	1578.3	4.7	0.183	0.214	0.105	0.239
2.25-2.13	2799	1397	2	56.19	1484	4.2	0.18	0.218	0.119	0.16
2.13-2.04	2063	1182	1.75	47.82	1407.8	3.9	0.167	0.204	0.114	0.196
2.04-1.96	1557	1058	1.47	42.7	1346.5	3.6	0.151	0.195	0.121	0.142
1.96-1.89	1204	899	1.34	36.06	1284.7	3.4	0.157	0.211	0.139	-0.081
14.42-1.89	57581	16199	3.55	64.32	3281.4	8.5	0.173	0.191	0.075	0.949

Table S5 Shell-by-shell merging statistics for PsbO sorted by I/σ(I).

Resolution	Num Obs	Num Unique	Multiplicity	Completeness	Mean I	Mean I/σ(I)	R_{merge}	R_{meas}	R_{pim}	$CC_{1/2}$
**Profile Fitted – Low I/σ(I)**										
11.46-4.64	821	346	2.37	31.6	885.7	2.9	0.433	0.522	0.281	0.194
4.64-3.73	1366	454	3.01	44.73	1067.8	3.3	0.343	0.397	0.191	0.174
3.73-3.27	2707	671	4.03	67.44	948.3	3.7	0.302	0.339	0.148	0.303
3.27-2.98	3875	802	4.83	81.59	872.8	4	0.299	0.33	0.133	0.4
2.98-2.77	4706	866	5.43	89	808.8	4.1	0.298	0.326	0.126	0.318
2.77-2.60	4514	866	5.21	90.3	746.5	3.9	0.302	0.331	0.131	0.254
2.60-2.48	4465	868	5.14	90.61	717.8	3.8	0.307	0.338	0.136	0.221
2.48-2.37	4364	870	5.02	91.39	701.1	3.7	0.31	0.344	0.142	0.113
2.37-2.28	4218	895	4.71	93.04	699.5	3.5	0.305	0.34	0.144	0.201
2.28-2.20	3989	860	4.64	92.18	697.7	3.4	0.306	0.342	0.147	0.222
11.46-2.20	35025	7498	4.67	76.3	791.4	3.7	0.308	0.343	0.143	0.293
**Profile Fitted – High I/σ(I)**										
13.98-4.69	8311	901	9.22	82.13	9418.1	40	0.204	0.216	0.066	0.913
4.69-3.75	10500	895	11.73	88.18	4995.3	33.5	0.216	0.224	0.058	0.949
3.75-3.28	7837	774	10.13	77.48	2700.3	19.5	0.244	0.254	0.064	0.866
3.28-2.98	5484	643	8.53	65.28	2143.3	13.9	0.254	0.265	0.069	0.776
2.98-2.77	3200	502	6.37	51.97	1761.9	10.1	0.237	0.251	0.073	0.582
2.77-2.61	1908	379	5.03	38.95	1654.3	8.4	0.221	0.237	0.078	0.465
2.61-2.48	1210	308	3.93	32.29	1579.2	7.2	0.216	0.236	0.087	0.545
2.48-2.37	979	259	3.78	27.47	1543.6	6.9	0.222	0.242	0.088	0.577
2.37-2.28	745	190	3.92	19.81	1505.3	6.9	0.214	0.232	0.081	0.517
2.28-2.20	380	132	2.88	14.16	1468.8	5.8	0.189	0.212	0.092	0.277
13.98-2.20	40554	4983	8.14	50.69	3873.6	20.9	0.218	0.229	0.064	0.937
**Spherical - Low I/σ(I)**										
10.33-4.61	1346	500	2.69	46.38	1917.6	2.7	0.201	0.238	0.123	0.301
4.61-3.71	2694	687	3.92	67.82	1398.7	3.2	0.209	0.237	0.106	0.224
3.71-3.26	3825	797	4.8	80.51	1098.7	3.5	0.211	0.233	0.095	0.246
3.26-2.97	4564	868	5.26	88.39	944.3	3.6	0.217	0.239	0.094	0.221
2.97-2.76	4806	879	5.47	90.34	809.9	3.7	0.217	0.238	0.094	0.096

2.76-2.60	4194	863	4.86	89.99	728.6	3.4	0.216	0.24	0.099	0.065
2.60-2.47	3963	857	4.62	89.83	671.7	3.3	0.213	0.238	0.102	0.083
2.47-2.37	3765	856	4.4	89.92	632.8	3.2	0.211	0.237	0.103	0.027
2.37-2.28	3632	876	4.15	91.44	599	3.1	0.21	0.238	0.106	0.025
2.28-2.20	3237	839	3.86	90.22	577	3.0	0.209	0.239	0.111	0.013
10.33-2.20	36026	8022	4.49	81.93	882.8	3.3	0.212	0.237	0.101	0.7
**Spherical - High I/σ(I)**										
13.98-4.69	7933	929	8.54	84.3	10818	24.2	0.182	0.193	0.06	0.931
4.69-3.74	9176	909	10.09	89.29	5108.6	20.2	0.182	0.19	0.051	0.967
3.74-3.28	6750	853	7.91	84.88	2669.1	11.6	0.196	0.206	0.059	0.88
3.28-2.98	4755	760	6.26	77.16	1945.2	8.4	0.199	0.212	0.066	0.697
2.98-2.77	3074	718	4.28	73.64	1578	6.4	0.188	0.206	0.078	0.363
2.77-2.61	2185	681	3.21	70.13	1378.8	5.4	0.176	0.199	0.085	0.255
2.61-2.48	1727	625	2.76	65.24	1283	5.0	0.175	0.203	0.097	0.239
2.48-2.37	1515	611	2.48	64.32	1194	4.7	0.174	0.204	0.099	0.11
2.37-2.28	1325	571	2.32	59.23	1121.5	4.5	0.166	0.194	0.095	0.151
2.28-2.20	1113	558	1.99	59.55	1055.8	4.1	0.162	0.198	0.11	-0.117
13.98-2.20	39553	7215	5.48	73.12	3226.8	10.6	0.184	0.195	0.06	0.96

Table S6 Shell-by-shell refinement statistics for the E166Q β-lactamase mutant.

	Profile Fitted				Spherical			
Mean Resolution (A)	CC_{work}	CC_{free}	R_{work}	R_{free}	CC_{work}	CC_{free}	R_{work}	R_{free}
9.42	0.90	0.92	0.19	0.19	0.91	0.92	0.18	0.18
3.65	0.87	0.87	0.21	0.24	0.86	0.85	0.21	0.24
3.04	0.77	0.73	0.25	0.30	0.71	0.66	0.27	0.33
2.70	0.79	0.70	0.24	0.28	0.69	0.58	0.28	0.32
2.48	0.77	0.65	0.24	0.27	0.62	0.56	0.29	0.30
2.32	0.74	0.67	0.25	0.28	0.53	0.40	0.29	0.33
2.20	0.67	0.61	0.27	0.28	0.42	0.43	0.32	0.32
2.09	0.62	0.45	0.30	0.36	0.36	0.25	0.35	0.38
2.01	0.49	0.31	0.34	0.37	0.25	0.13	0.37	0.38
1.93	0.36	0.19	0.36	0.43	0.17	0.14	0.38	0.42

Table S7 Shell-by-shell refinement statistics for the PsbO.

	Profile Fitted				Spherical			
Mean Resolution (A)	CC_{work}	CC_{free}	R_{work}	R_{free}	CC_{work}	CC_{free}	R_{work}	R_{free}
9.33	0.83	0.73	0.27	0.31	0.81	0.70	0.27	0.32
4.21	0.89	0.88	0.21	0.21	0.88	0.84	0.22	0.24
3.51	0.79	0.83	0.25	0.26	0.79	0.79	0.27	0.29
3.13	0.79	0.81	0.23	0.27	0.70	0.73	0.26	0.31
2.87	0.72	0.59	0.26	0.29	0.56	0.47	0.28	0.30
2.69	0.66	0.53	0.27	0.31	0.48	0.41	0.29	0.35
2.54	0.60	0.60	0.29	0.34	0.45	0.47	0.30	0.37
2.42	0.57	0.53	0.29	0.39	0.44	0.44	0.31	0.36
2.32	0.53	0.16	0.31	0.38	0.38	0.39	0.33	0.39
2.24	0.42	0.42	0.31	0.33	0.30	0.34	0.34	0.40

Table S8 Shell-by-shell refinement statistics for the PaPth1.

	Profile Fitted				Spherical			
Mean Resolution (A)	CC_{work}	CC_{free}	R_{work}	R_{free}	CC_{work}	CC_{free}	R_{work}	R_{free}
9.61	0.72	0.80	0.31	0.32	0.71	0.81	0.26	0.32
4.95	0.88	0.82	0.22	0.26	0.87	0.76	0.20	0.27
4.14	0.86	0.70	0.22	0.31	0.84	0.63	0.21	0.29
3.69	0.81	0.63	0.25	0.30	0.72	0.39	0.27	0.40
3.39	0.71	0.42	0.28	0.42	0.60	0.37	0.30	0.43
3.17	0.75	0.19	0.26	0.44	0.43	0.24	0.38	0.37
3.00	0.70	0.56	0.28	0.38	0.36	0.28	0.36	0.42
2.86	0.64	0.48	0.29	0.34	0.35	-0.10	0.37	0.42
2.75	0.61	0.39	0.27	0.36	0.20	0.10	0.40	0.44
2.65	0.55	0.17	0.31	0.39	0.22	0.01	0.39	0.41

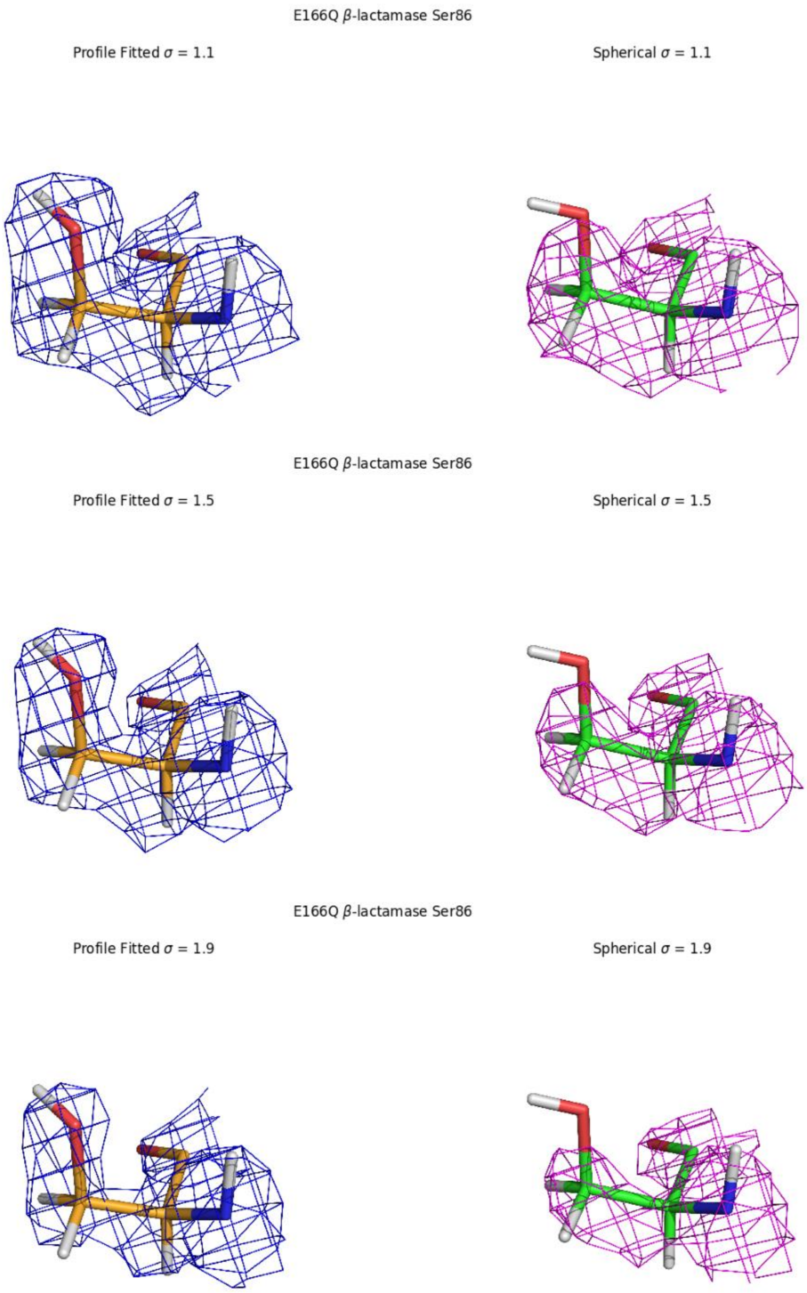

Figure S1 2mFo-DFc density maps of E166Q β-lactamase Ser86 at (from top to bottom) 1.1σ, 1.5σ, 1.9σ. Profile fitted maps are on the left and spherical maps are on the right.

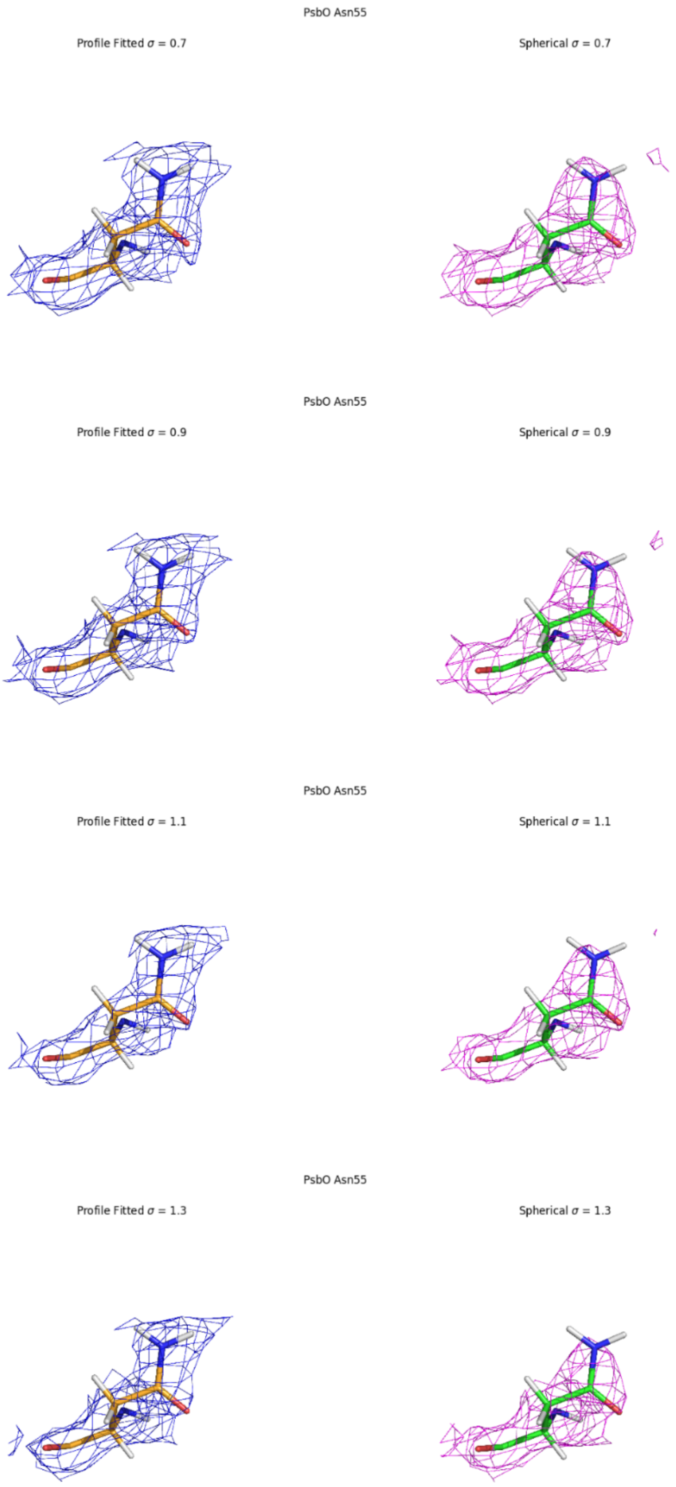

Figure S2 2mFo-DFc density maps of PsbO Asn55 at (from top to bottom) 0.7σ, 09σ, 1.1σ, 1.3σ. Profile fitted maps are on the left and spherical maps are on the right.

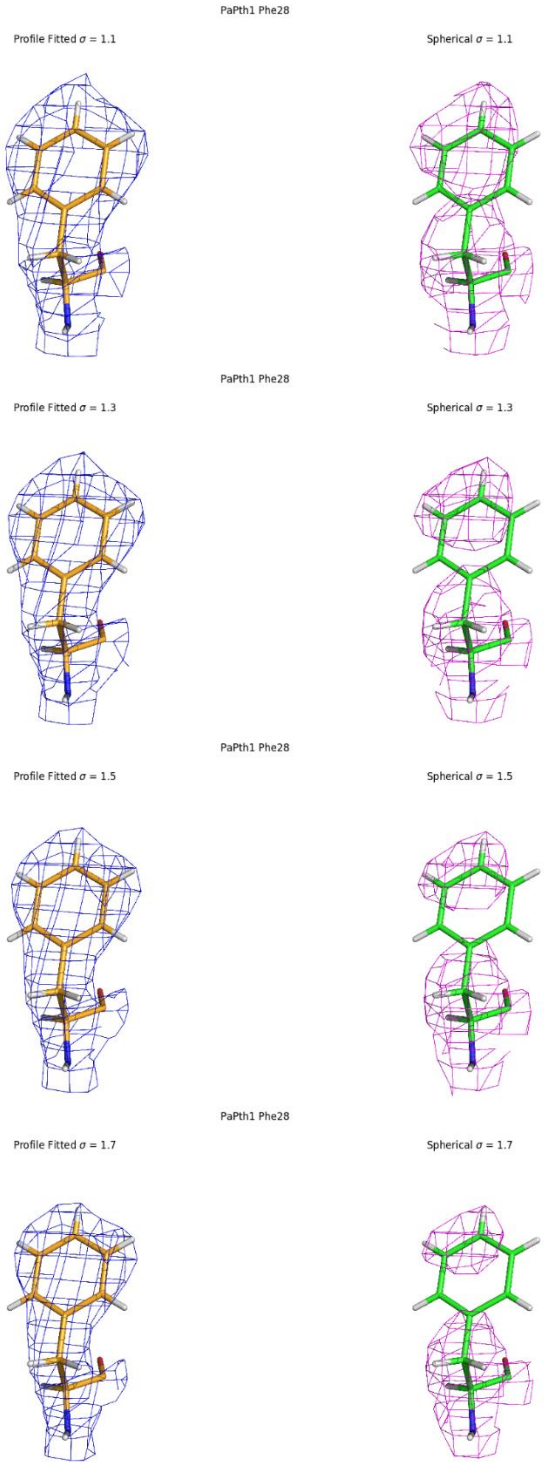

Figure S3 2mFo-DFc density maps of PaPth1 Phe28 at (from top to bottom) 1.1σ, 1.3σ, 1.5σ, 1.9σ. Profile fitted maps are on the left and spherical maps are on the right.
